# Cryopreservation of dehydrated *Caenorhabditis elegans* with multiple recoveries using a granular medium

**DOI:** 10.17912/micropub.biology.000488

**Published:** 2021-10-25

**Authors:** Julia Hayden, Christopher Fang-Yen

**Affiliations:** 1 Department of Bioengineering, University of Pennsylvania

## Abstract

Ultracold storage is widely used to preserve genetic stocks. Standard cryopreservation methods for the nematode *C. elegans* are vulnerable to refrigeration failures, which can result in the loss of stock viability due to freeze-thaw damage. In previous work our laboratory developed a method for cryopreserving worms in a dehydrated form that remains viable after multiple freeze-thaw cycles. However, strains preserved in this manner can be recovered only once from each cryopreservation tube. Here we describe a cryopreservation method in which *C. elegans* are dehydrated in a granular medium (cornmeal) prior to freezing. To recover worms, a small fraction (~1%) of the medium may be removed with the remainder returned to cold storage. Our improved cryopreservation method is not only resistant to refrigeration failures but also greatly increases the number of recoveries per tube compared to current methods.

**Figure 1. Worms about 1 h after recovery on an OP50-seeded NGM plate:**
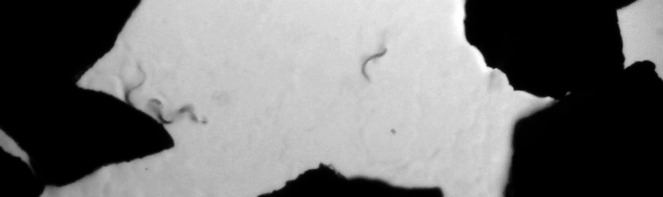
Dark shapes are cornmeal granules.

## Description

One advantage of *C. elegans* as an experimental model is its ability to be indefinitely preserved at ultracold temperatures. Two solutions are commonly used for cryopreservation of *C. elegans*. The liquid freezing buffer (Brenner 1974) contains glycerol as cryoprotectant. Since worms frozen with this solution settle to the bottom of the cryotube, recovering them requires thawing the entire tube. As a result, only one recovery is possible per tube. In the soft agar method developed by Leon Avery (Stiernagle 2006), the freezing solution contains agar in addition to glycerol. The agar gel keeps worms suspended throughout the tube during freezing. This allows a small portion of the tube to be scooped out for recovery, with the remainder returned to the freezer without thawing. Worms can typically be recovered 3-4 times from 1 mL of soft agar (Stiernagle 2006). The ability to perform several recoveries per tube reduces the need to prepare and store multiple tubes for cryopreservation.

In commonly used methods for cryopreservation, stocks are vulnerable to refrigeration failures due to freeze-thaw damage. Our group recently developed a method for cryopreserving worms in a dehydrated form that remains viable after multiple freeze-thaw cycles (McClanahan *et al.* 2020). Dehydration of worms in a protectant (glycerol or trehalose) prior to freezing prevented damage during refreezing. However, in this method the entire vial must be rehydrated to recover the strain.

Here we present an improved dry freezing method that allows for multiple recoveries per vial. We reasoned that allowing worms to dehydrate in a granular medium would make them easier to separate after drying, since worms would adhere to the granules throughout the tube instead of to the tube or to one another. We prepared 0.6 g cornmeal (dried and ground *Zea mays*) in each cryotube, added a suspension of worms in a glycerol freezing solution, and mixed briefly. The mixture was allowed to dehydrate for 48 h before freezing. To recover the strain, we removed a small quantity of granules (5-10 mg) from the cryotube and added directly to an OP50-seeded plate. We observed worms feeding on the bacteria within 1-2 h (Fig. 1). Based on the total mass of cornmeal and dehydrated worms, we estimate that worms can be recovered from a single tube 100-200 times. We did not quantify survival rates or long-term viability under ultracold storage but expect them to be similar to that of worms desiccated without the granular medium.

As in the previous study, we would expect that *daf-16* mutants, and possibly other dauer-defective strains, cannot be preserved by this method since they did not remain viable after desiccation (McClanahan *et al.* 2020).

These results show that dry freezing with a granular medium is an effective way of preserving strains in a manner that is not only resistant to refrigeration failure but also allows a large number of recoveries per tube. This method has the potential to reduce the manual effort required for freezing multiple tubes and allow for more efficient use of ultracold storage space.

## Methods

***C. elegans strains and maintenance***

Experiments were performed with the Bristol N2 strain. We cultured *C. elegans* on OP50 *E. coli* using standard methods (Stiernagle 2006).

***Dry freezing C. elegans in a granular medium***

We picked five gravid adult hermaphrodites onto each of two 6 cm seeded plates and allowed the population to grow at room temperature (18-22° C) for three weeks. At this point the plates contain a mixed stage population dominated by L1-L3 larvae with a small number of dauer animals (McClanahan *et al.* 2020). We washed worms using S buffer (Stiernagle 2006) into a 15 mL conical centrifuge tube and added S buffer to a final volume of 10 mL. We centrifuged for 5 minutes at 700 rcf to pellet worms, removed supernatant, added fresh S buffer to 10 mL, pelleted worms again, and removed supernatant again, leaving < 100 µL of S buffer with the worms. We transferred worms to a 1.5 mL centrifuge tube and added S buffer to a final volume of 125 µL. We then added 125 µL of worm freezing buffer (30% glycerol in S buffer) and mixed by gentle vortexing or flicking the tube.

We added 0.6 g of heat-sterilized cornmeal (Bob’s Red Mill Medium Grind 1148S24) to a 1.8 mL volume cryotube. We pipetted 200-250 µL of the worm mixture into the tube and mixed with the pipette tip.

To dehydrate worms, we followed a procedure similar to that previously described (McClanahan *et al.* 2020). We placed the uncapped cryotube upright into a tube rack inside an airtight plastic box containing 20 g desiccant (anhydrous calcium sulfate with cobalt chloride indicator, 8 mesh size, Drierite, W.A. Hammond). After 48 h, tubes were capped and transferred to a -80 ºC freezer.

To recover worms, we used a spatula to remove a small sample of cornmeal grains and placed them directly on an OP50-seeded NGM plate. A 10 mg sample typically yielded 20-30 recovered animals.
